# Rice Genome Approaches Completion

**DOI:** 10.1371/journal.pbio.0030047

**Published:** 2005-02-01

In April 2002, *Science* published draft genome sequences for the two major subspecies of cultivated rice, Oryza sativa. The release of the rice genome—the first plant crop to be sequenced—was big news. Rice is a staple crop for more than half of the world's population, and it was hoped that the availability of the genome sequence might enable scientists to develop more productive rice strains or strains that are more environmentally friendly. Furthermore, the rice genome might provide the key to understanding the genetics of other major cereal crops, all of which have much larger genomes.

But the sequences published in 2002 were only draft genomes, containing many gaps and errors—works-in-progress rather than finished products. Now, a large group of scientists led by the Beijing Institute of Genomics is publishing a much improved, near-complete genome analysis of the *indica* and *japonica* subspecies of O. sativa, which are eaten in India and China, and Japan, respectively. Their analysis team, led by Gane Ka-Shu Wong, provides important insights into the evolution of rice.

First of all, the team improved their original whole-genome shotgun sequencing of *indica* by generating significantly more sequence data, and then they used better methods to assemble these data. In whole-genome shotgun sequencing, the entire genome is chopped into random fragments, each fragment is sequenced, and then powerful computer programs search for overlaps and put all these data in order. It's like putting a fiendishly difficult jigsaw puzzle together by looking for patches of matching color.

The key to the improvement in the genome sequence analysis is that the researchers have used the combined DNA sequence data from the two subspecies to facilitate the sequence assembly. The result is a nearly 1,000-fold increase in contiguity for the two genome sequences. In other words, while the original draft was very fragmented, in the new version, 97.7% of the genes can be found, in either the *indica* or the *japonica* dataset, on one piece of DNA whose position along the chromosomes is well defined.

The researchers have also used their improved genome sequence to investigate the evolutionary history of rice. Central to evolution is the development of new functions through mutation of existing genes. But when mutations occur in functional genes, the result is rarely beneficial, so it is thought that evolution is more likely to proceed first by duplicating existing genes and then experimenting on the “backup” copy of the gene. Wong and colleagues report that there is evidence in the rice DNA sequences for a whole-genome duplication event just before the grasses diverged from other flowering plants, about 55–70 million years ago. This genome duplication may have played a role in the origin of the grasses, which then spread rapidly across the world to provide important sources of food that, among other things, possibly influenced human evolution.[Fig pbio-0030047-g001]

**Figure pbio-0030047-g001:**
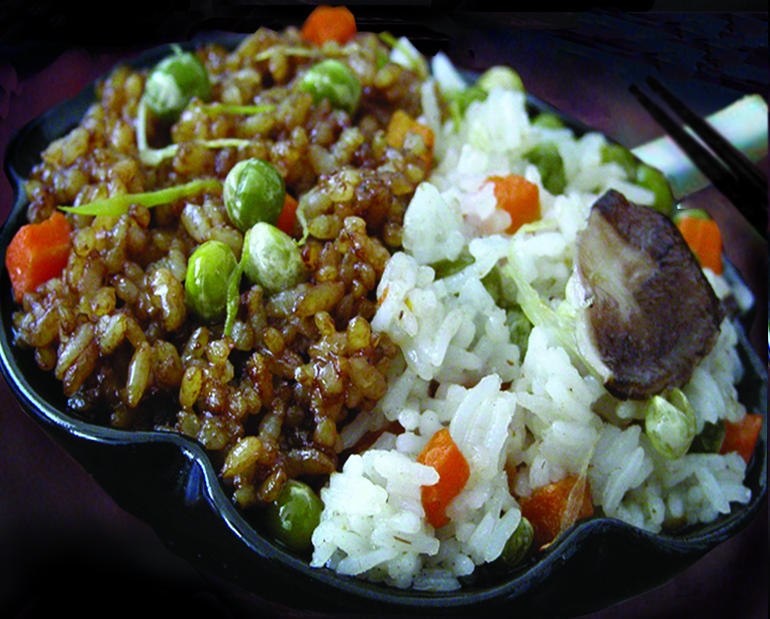
A bowl of *indica* (white, long grains) and *japonica* (brown, short grains) rice

Analysis of the rice genomes also indicates that a small chromosomal segment was duplicated about 21 million years ago and that there is massive ongoing duplication of individual genes. These individual gene duplications provide a continuous source of raw material for gene genesis and very likely contribute to the differences between members of the grass family. Now the challenge is to use the rice sequences as a basis for detailed genetic analyses of additional cereal crops and for the development of improved strains of not only rice, but wheat, maize, and other important food crops.

